# Esomeprazole inhibits proliferation of scleroderma fibroblasts via cell cycle regulation

**DOI:** 10.3389/fphar.2025.1703115

**Published:** 2026-01-06

**Authors:** Mohammad A. Khan, Shaheer Koniyan, Manisha Ahir, Anil G. Jegga, Maureen D. Mayes, David Gius, Justin Leung, Mark D. Bonnen, Sydney B. Montesi, Yohannes T. Ghebre

**Affiliations:** 1 Department of Radiation Oncology, The University of Texas Health Science Center at San Antonio, San Antonio, TX, United States; 2 Department of Radiation Oncology, Baylor College of Medicine, Houston, TX, United States; 3 Division of Biomedical Informatics, Department of Pediatrics, Cincinnati Children’s Hospital Medical Center, University of Cincinnati College of Medicine, Cincinnati, OH, United States; 4 Department of Internal Medicine, Division of Rheumatology, UTHealth McGovern Medical School, Houston, TX, United States; 5 Mays Cancer Center, The University of Texas Health Science Center at San Antonio, San Antonio, TX, United States; 6 Barshop Institute for Longevity and Aging Studies, University of Texas Health San Antonio, Antonio, TX, United States; 7 Department of Radiation Oncology, Tufts University School of Medicine, Boston, MA, United States; 8 Division of Pulmonary and Critical Care Medicine, Massachusetts General Hospital, Boston, MA, United States

**Keywords:** esomeprazole, fibroblast, proliferation, scleroderma, CDKs, skin fibrosis

## Abstract

**Background:**

Scleroderma is a complex autoimmune disease characterized by abnormal fibroblast proliferation and excessive collagen deposits in the skin and internal organs. We previously showed that esomeprazole, an FDA-approved drug for gastric disorders, may prevent dermal fibrosis.

**Methods:**

To test this, we evaluated the antiproliferative effect of esomeprazole and the underlying molecular mechanisms using primary fibroblasts derived from patients with scleroderma. BrdU incorporation, flow cytometry, immunofluorescence, Western blot analysis, RNA sequencing, and functional enrichment analysis were performed.

**Results:**

Esomeprazole inhibited the proliferation of scleroderma fibroblasts in a dose-dependent manner, as measured by BrdU incorporation and Ki-67 marker. Intriguingly, esomeprazole arrested fibroblasts in the G1 phase of the cell cycle, resulting in a reduction of cells in the S phase. Expression of p21, a known inhibitor of cyclin-dependent kinases (CDKs), was elevated, while CDK1 and CDK2 levels were decreased following esomeprazole treatment.

**Discussion:**

These results suggest that esomeprazole induces G1 phase arrest by upregulating p21 and downregulating CDK1 and CDK2, thereby inhibiting fibroblast proliferation. These data provide important insights into how esomeprazole regulates fibroblast proliferation in scleroderma and suggest that it may represent a potential therapeutic intervention.

## Introduction

Scleroderma is a rare autoimmune disease thought to be induced by environmental triggers genetically susceptible individuals, leading to the recruitment of inflammatory cells, uncontrolled proliferation of fibroblasts, and differentiation into collagen-synthesizing myofibroblasts (Singh et al., 2019; Gabrielli et al., 2009; Brown and O'Reilly, 2019). While localized scleroderma is typically restricted to small areas of the skin, systemic sclerosis involves widespread microvascular damage and profound fibrosis of multiple organs, includeing the skin and lungs (Denton and Khanna, 2017; Khanna et al., 2015).

Despite advances in understanding pathogenic drivers of scleroderma, mortality remains high, and 5-year survival rates are worse than those of many cancers (Pokeerbux et al., 2019; Rubio-Rivas et al., 2014). While there are limited treatment options to date, including immunomodulatory drugs such as cyclophosphamide (Tashkin et al., 2006) and mycophenolate (Derk et al., 2009), these therapies are unable to reverse established fibrosis or cure the disease. Thus, there is a critical need to develop novel antifibrotic therapies for the treatment of scleroderma.

In this regard, proton pump inhibitors (PPIs), an FDA-approved class of drugs for the treatment of gastroesophageal reflux disease (GERD), have recently been linked to a range of extra-intestinal biological activities, including direct modulation of fibroblast overproliferation in response to transforming growth factor beta (TGF-β) (Ghebremariam et al., 2015; Hammond et al., 2019; Ebrahimpour et al., 2022), as well as attenuation of tissue inflammation (Kedika et al., 2009; Namazi and Jowkar, 2008; Handa et al., 2006; Yoshida et al., 2000) and fibrosis (Ghebremariam et al., 2015; Ebrahimpour et al., 2022; Ghebre YT. and Raghu G., 2016; Ghebre Y. and Raghu G., 2016). Among the PPIs, we found esomeprazole to be the most potent antifibrotic molecule and capable of modulating several profibrotic cytokines, fibroblast-to-myofibroblast transdifferentiation, and collagen deposition both *in vitro* and in animal models of damage-induced lung remodeling (Ghebremariam et al., 2015; Ebrahimpour et al., 2021; Nelson et al., 2017; Pham et al., 2019). Accordingly, we introduced the concept of repurposing esomeprazole as an antifibrotic drug for the treatment of lung fibrosis and other diseases characterized by abnormal deposition of extracellular matrix proteins (ECMs), including scleroderma (Ghebre YT. and Raghu G., 2016).

More recently, we formulated esomeprazole into a topical product and demonstrated its efficacy in mitigating radiation-induced skin inflammation and fibrosis (Pham et al., 2019). In this study, we investigated whether esomeprazole inhibits the proliferation of fibroblasts derived from scleroderma patients with limited disease (SSc-limited). To understand the underlying molecular mechanisms, we investigated the effect of esomeprazole on cell cycle arrest and on the expression of key cell cycle-regulating proteins, including p21 and cyclin-dependent kinases (CDK1 and CDK2). CDK1 and CDK2 are critically crucial for fibroblast progression through the cell cycle. CDK2 is critical for the G1-S phase transition, while CDK1 plays an important role in the G2-M phase of the cell cycle (Uxa et al., 2021; Gartel et al., 1996; Schafer, 1998). Collectively, dysregulation of these proteins has been associated with the uncontrolled proliferation of fibroblasts and the abnormal deposition of ECM proteins, including collagen and fibronectin. In this study, we used molecular and cell biological tools to specifically understand how esomeprazole influences the proliferation of scleroderma fibroblasts.

## Methods

### Cell culture

Dermal fibroblasts were isolated under consent from de-identified skin biopsies of scleroderma patients diagnosed with limited disease as described (Gardner et al., 2006). The cells were cultured in Dulbecco’s Modified Eagle medium (DMEM; Gibco, cat # 11995) supplemented with 10% fetal bovine serum (FBS; Gibco, cat # A5256801) and 1% penicillin-streptomycin (Gibco, cat # 15140122), and maintained at 37 °C in 5% CO_2_. The experiments were performed using low passage number cells (p < 6) to maintain phenotypic consistency. Fibroblast cell identification/purity was analysed using fibroblast-specific protein antibody S1004A/FSP1(66489-1-Ig, Proteintech) (Supplementary Figure S1).

### BrdU incorporation assay

To evaluate proliferation, 2.5 × 10^3^ cells per well were seeded in 96-well plates and cultured for 24 h. The following day, the cells were serum-starved for 2 h, followed by maintenance in low-serum (0.1% fetal bovine serum, or FBS) conditions for an additional 22 h to synchronize the cells. Esomeprazole sodium (Tecoland, batch # 20240126) was used at final concentrations ranging from 10 to 100 µM for 24 h. Bromodeoxyuridine (BrdU) (Sigma, cat # 2750) was incorporated into proliferating cells following 16 h of treatment with the molecule before fixation. Detection was performed using an anti-BrdU antibody, followed by spectrophotometric quantification at optical density (OD) 450 nm (SpectraMax iD3, Molecular Devices, United States).

### Flow cytometry for cell cycle analysis

Synchronized cells were treated with esomeprazole at concentrations of 50 µM or 100 µM for 24 h. The cells were then dissociated using Accutase (ThermoFisher; cat # A1110501), washed with phosphate-buffered saline (PBS), and fixed in 75% ethanol at −20 °C overnight. The next day, the cells were washed with PBS and resuspended in a staining solution containing propidium iodide (5 μg/mL) and RNase A (50 μg/mL), followed by incubation at 37 °C for 30 min. Flow cytometry was performed using the BD LSR II system (BD Biosciences, United States), and the distribution of cells across the G1, S, and G2/M phases was quantified using FlowJo software.

### Immunofluorescence assay for Ki-67 expression

Cells were seeded onto Fluoro Dishes (World Precision Instruments, cat # FD3510100) and were allowed to adhere to for 24 h prior to synchronization. After treatment with esomeprazole (50 and 100 µM) for 24 h, the cells were fixed using 4% paraformaldehyde for 15 min at room temperature. Permeabilization was performed using 0.2% Triton X-100, followed by blocking with 2.5% bovine serum albumin (BSA). For immunostaining, the cells were incubated overnight with Ki-67- D3B5 (Cell Signaling Technology; CST, cat #9129S) at a dilution of 1:800). After washing, the cells were incubated with Alexa Fluor 647-conjugated goat anti-rabbit IgG (at a dilution of 1:1,000) to complete the staining process. Actin filaments were stained with Phalloidin-Alexa Fluor 488 (at a dilution of 1:1,000), and nuclei were counterstained with 4′,6-diamidino-2-phenylindole (DAPI) (Sigma, cat #D9542). Images were captured using a Leica Stellaris 5 confocal microscope.

### Western blot analysis

Protein was isolated from the cells using RIPA buffer and separated using Sodium Dodecyl Sulfate Polyacrylamide Gel Electrophoresis (SDS-PAGE). The separated protein was transferred onto a membrane using the iBLOT2 transfer system (Invitrogen, United States). The membrane was blocked using a 5% milk solution prepared in Tris Buffered Saline with Tween-20 (TBST) and incubated overnight at 4 °C with the following primary antibodies: p21 (CST, cat # 2947S), CDK1 (Proteintech, cat # 67575-1), and CDK2 (Proteintech, cat # 60312-1). β-Actin (CST, cat# 4967) was used as an internal control at a dilution of 1:5,000. After washing, the membrane was incubated with Horseradish Peroxidase (HRP)-conjugated secondary antibodies. Protein bands were visualized using enhanced chemiluminescence (ECL) reagent and detected using the FluorChem E imaging system (Protein Simple, United States). Densitometric analysis was performed using ImageJ software.

### RNA-seq and bioinformatics analysis

RNA sequencing was performed using Illumina NovaSeq 100PE sequencing following library preparation with a stranded mRNA-seq library preparation kit. Quality control ( Q30 score >90% and removal of reads <20) was performed using FastQC, followed by read alignment to the human reference genome (GRCh38) using the STAR aligner. Differentially expressed genes (DEGs) were subsequently identified using the DESeq2 tool with the following two criteria: FDR-adjusted p-value < 0.05 and fold change (in either way) > 0.58. To further analyze the biological significance of the DEGs, functional enrichment analysis was performed using the ToppFun application of the ToppGene suite (Chen et al., 2009) and the results were visualized using Cytoscape (version 3.10.3) (Shannon et al., 2003).

### Statistical analysis

All experiments were conducted at least in triplicate to ensure reproducibility. Statistical comparisons were performed using one-way Analysis of Variance (ANOVA), with the Bonferroni *post hoc* test applied for multiple comparisons. A p-value of less than 0.05 (p < 0.05) was considered statistically significant. Results are presented as mean ± standard error of the mean (SEM) and were analyzed using GraphPad Prism software, version 10.4.0.

## Results

### Esomeprazole inhibits proliferation of scleroderma fibroblasts

**FIGURE 1:**
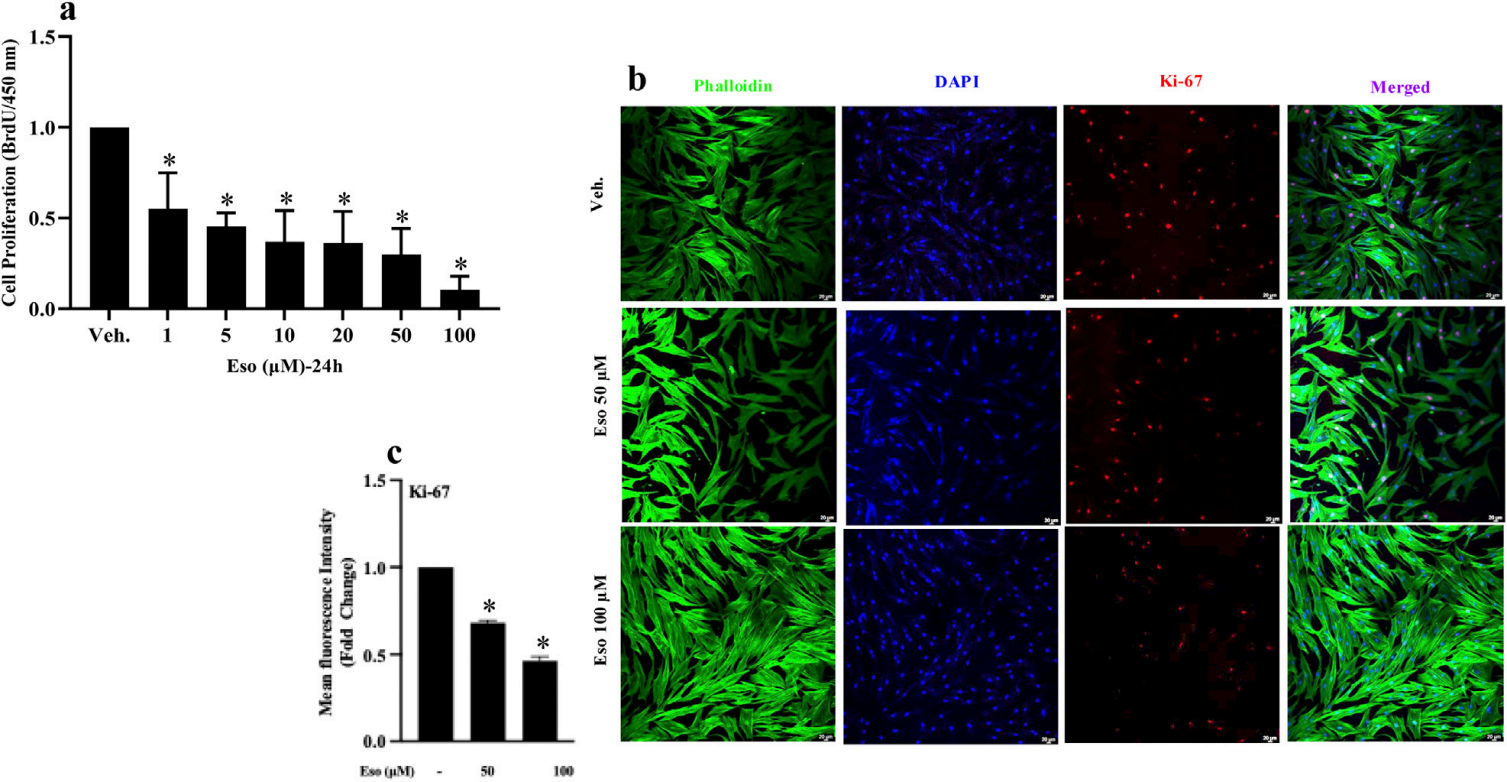
Effect of esomeprazole on proliferation of scleroderma fibroblasts Scleroderma fibroblasts were cultured with increasing concentrations of esomeprazole (1–100 µM) for 24 h. Cell proliferation was assessed using **(a)** the BrdU incorporation assay, and **(b,c)** by immunofluorescence staining for Ki-67. Data represent mean ± SEM; N = 3. *p < 0.05.

Fibroblasts from scleroderma patients are characterized by excessive proliferation, contributing to fibrosis and pathological ECM remodeling. To evaluate the effect of esomeprazole on the proliferation of fibroblasts, a BrdU incorporation assay was performed as described above. Importantly, the study revealed that esomeprazole reduced the proliferation of scleroderma fibroblasts in a concentration-dependent manner, with an inhibition of approximately 85% at the highest concentration (100 µM) (p < 0.05, N = 3) compared to the vehicle-treated control group (Figure 1a).

To further confirm these findings, immunofluorescence staining for Ki-67 was performed, an established biomarker of proliferation (Uxa et al., 2021). Here, the data (Figures 1b,c) demonstrate a significant reduction in Ki-67 expression in cells treated with esomeprazole. Importantly, the treatment did not induce any noticeable cell abnormalities or death, as evidenced by the retention of normal cellular morphology (Gardner et al., 2006). Taken together, these findings indicate that esomeprazole effectively suppresses the proliferation of scleroderma fibroblasts.

### Esomeprazole modulates key cell cycle regulatory proteins to induce G1 phase

**FIGURE 2:**
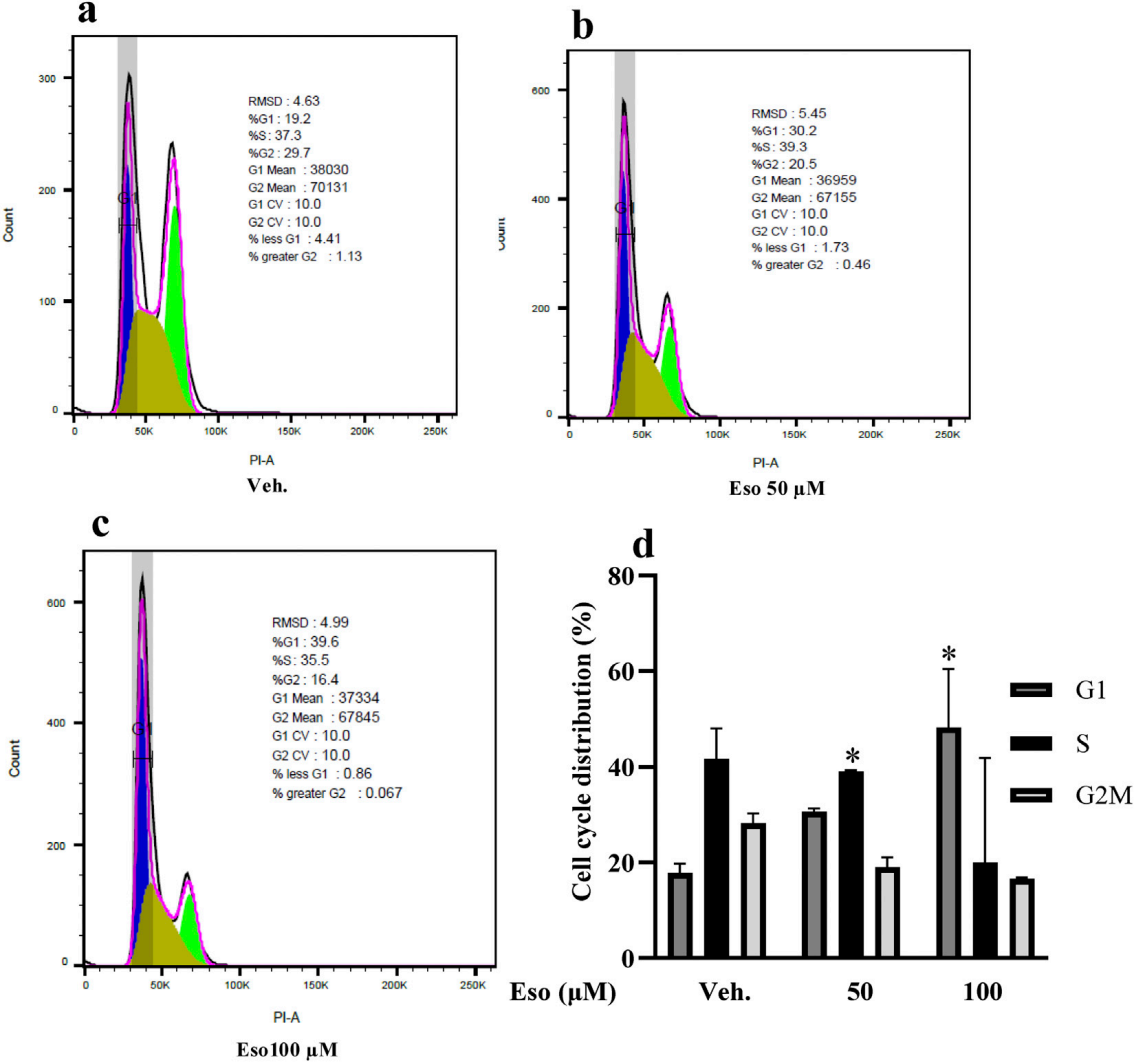
Esomeprazole Modulates Key Cell Cycle Regulatory Proteins to Induce G1 Phase Scleroderma patient-derived fibroblasts were cultured and treated with esomeprazole (50–100 µM) for 24 h. Cell cycle distribution was analyzed by flow cytometry. **(a–c)** Representative data for Vehicle, 50 μM, and 100 µM esomeprazole treatment. **(d)** Quantification of cell cycle phase distribution. Data represent mean ± SEM; N = 3. *p < 0.05.

To determine the mechanism by which esomeprazole inhibits the proliferation of scleroderma fibroblasts, we studied the cell cycle using flow cytometry. Intriguingly, the findings demonstrated a significant accumulation of cells in the G1 phase following esomeprazole treatment (Figures 2a–d). At 100 μM, for example, approximately 65% of cells were arrested in the G1 phase (p < 0.05), accompanied by a notable reduction in the number of cells progressing to the S and G2/M phases of the cell cycle. These results suggest that esomeprazole limits the G1-to-S phase transition, thereby limiting scleroderma fibroblast proliferation.

### Esomeprazole modulates key cell cycle regulatory proteins

**FIGURE 3:**
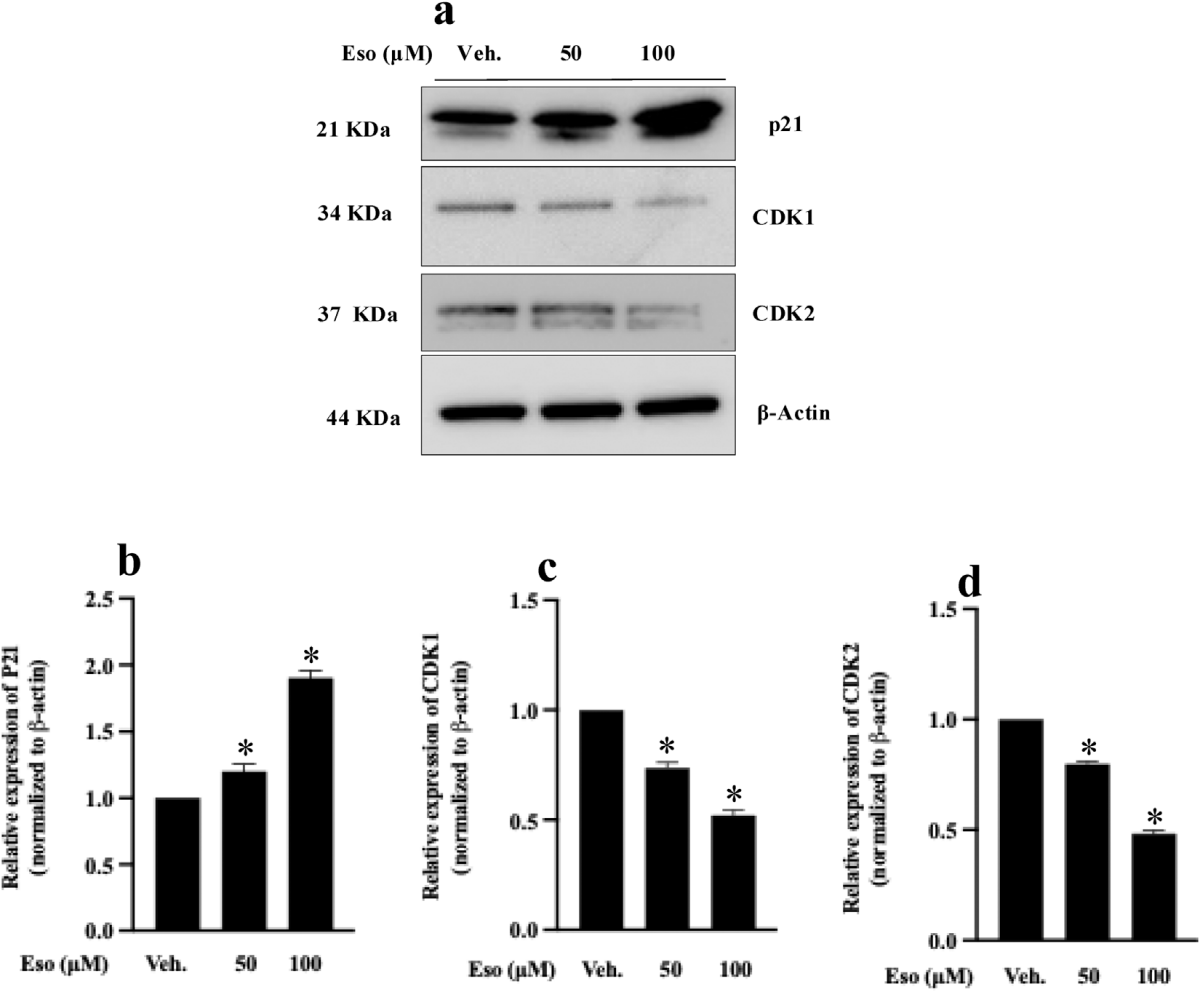
Effect of esomeprazole on the expression of p21 and CDK proteins Scleroderma patient-derived fibroblasts were cultured and treated with esomeprazole (50–100 µM) for 24 h **(a–d)** Representative blots are shown alongside quantification of protein expression levels (normalized to β-actin). Data represent mean ± SEM; N = 3. *p < 0.05.

To elucidate the molecular mechanism(s) underlying G1 phase arrest, we examined the expression of key cell cycle regulatory proteins, including p21, CDK1, and CDK2, using Western blot analysis. These results (Figure 3a) showed that treatment with esomeprazole resulted in a significant upregulation (1.5-fold) of p21 expression compared to control cells (p < 0.05) (Figure 3b). Concurrently, CDK1 and CDK2 expression levels were also downregulated by approximately 1.3-fold (Figures 3c,d), corroborating the observed G1 phase arrest by flow cytometry. Notably, the increased expression of p21 observed in this study indicates that esomeprazole enforces tighter control of the G1 checkpoint by acting on the p21 protein to limit the number of cells that cycle through during proliferation.

### Transcriptomic analysis reveals downregulation of proliferation-associated genes

**FIGURE 4:**
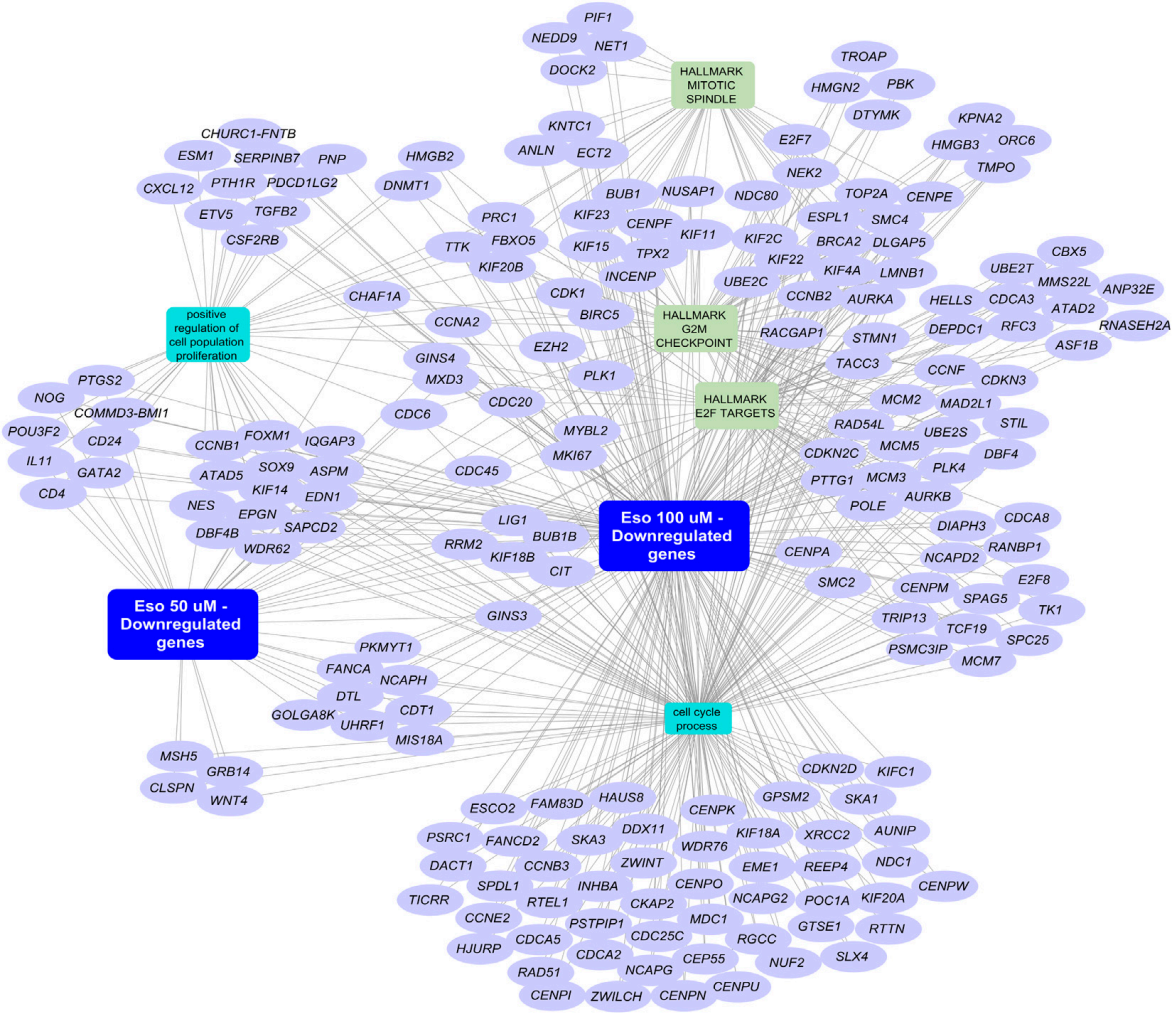
Network representation of select enriched biological processes among downregulated genes following esomeprazole treatment. Representative network map of selected biological processes of interest with accompanying genes. Purple nodes represent genes associated with cell proliferation and cell cycle regulation (rectangular nodes) that are downregulated in scleroderma fibroblasts treated with esomeprazole (50/100 µM) for 24 h. Functional enrichment was performed using the ToppFunn application of the ToppGene Suite. The network was generated using Cytoscape.

To further analyze the biological processes and pathways regulated by esomeprazole, RNA sequencing of scleroderma fibroblasts following esomeprazole treatment was undertaken. Intriguingly, differential gene expression analysis revealed the downregulation of 202 genes (Supplementary Table S1) associated with cell proliferation and cell cycle regulation (Figure 4). For example, treatment with esomeprazole was found to downregulate cell cycle regulatory genes, such as CDK1, cyclin B (CCNB), and Cell Division Cycle Protein 20 (CDC20). These transcriptomic findings indicate that esomeprazole exerts a pleiotropic effect on the biology of fibroblast proliferation, involving several interrelated molecular pathways.

## Discussion

Scleroderma remains a disease of high morbidity and mortality because of its complex pathophysiology and the paucity of effective treatment options (Singh et al., 2019; Gabrielli et al., 2009; Pokeerbux et al., 2019; Rubio-Rivas et al., 2014). The disease process in scleroderma is typically characterized by chronic inflammation and progressive fibrosis, leading to tissue stiffening and significant functional impairment and organ failure (Denton and Khanna, 2017; Khanna et al., 2015). Our previous studies using animal models that manifest inflammatory and fibrotic changes have demonstrated that esomeprazole is effective in attenuating these pathological changes (Ebrahimpour et al., 2021; Pham et al., 2019). Despite these promising results, the specific molecular mechanism(s) underlying the antifibrotic properties of the drug remain unclear. In this study, we investigated the effects of esomeprazole on scleroderma fibroblast proliferation and the mechanism by which the drug controls cell proliferation.

Fibroblasts are key cellular players in the pathobiology of scleroderma, as the only active cell population centrally involved in the turnover of ECM proteins and the progression of fibrosis (Singh et al., 2019; Gabrielli et al., 2009; Gillesberg et al., 2025). Our recent preclinical studies have shown that esomeprazole attenuates dermal fibrosis induced by ionizing radiation (Ebrahimpour et al., 2021; Pham et al., 2019). In the present study, we used the BrdU incorporation assay to provide direct evidence that esomeprazole effectively limits scleroderma fibroblast proliferation in a concentration-dependent manner (Figure 1a). To confirm this finding, we examined Ki-67, a classic regulator of cell proliferation (Uxa et al., 2021), and observed that esomeprazole significantly reduced its expression (Figures 1b,c). The BrdU assay measures DNA synthesis in proliferating cells, whereas the Ki-67 assay reflects the overall proliferative status of the cells (Gaglia et al., 1993). Together, these assays indicate that esomeprazole impacts both DNA synthesis (i.e., new cell formation) and cell cycle progression. This study aligns with previous reports describing the anti-proliferative effects of PPIs (Ebrahimpour et al., 2022; Hebert et al., 2022; Bernier et al., 2004).

Further mechanistic analysis of the cell cycle data revealed that esomeprazole arrests scleroderma fibroblasts at the G1 phase, resulting in a reduced proportion of cells in the S and G2/M phases of the cell cycle (Figures 2a–d). More specifically, esomeprazole disrupts the transition from the G1 to the S phase, thereby inhibiting subsequent cell division. These findings are consistent with our previous observations that esomeprazole induces G1 arrest in cancer cells (Hebert et al., 2021; Wang et al., 2021; Tozzi et al., 2020), leading to reduced DNA synthesis and downregulation of proliferation markers. Although studies report that esomprazole can trigger apoptosis or autophagy in cancer cells under inflammatory stress (Marino et al., 2010; Chueca et al., 2016; Gould et al., 2023; Du et al., 2022), our data did not show any apoptotic or autophagic features in scleroderma fibroblasts. At 50 and 100 μM concentrations of esomprazole, Ki-67 immunofluorescence showed fibroblasts remained healthy, demonstrating intact nuclear morphology (no nuclear fragmentation was observed) and preserved cytoskeleton (Figure 1b). In the context of scleroderma, esomeprazole slows the cell cycle progression, inhibits the fibroblasts proliferation without inducing cell death. Notably, pharmacological agents that inhibit the cell cycle have been shown to attenuate fibrotic changes (Gillesberg et al., 2025), further supporting the potential role of esomeprazole in modulating uncontrolled fibroblast proliferation and mitigating fibrosis progression.

**FIGURE 5:**
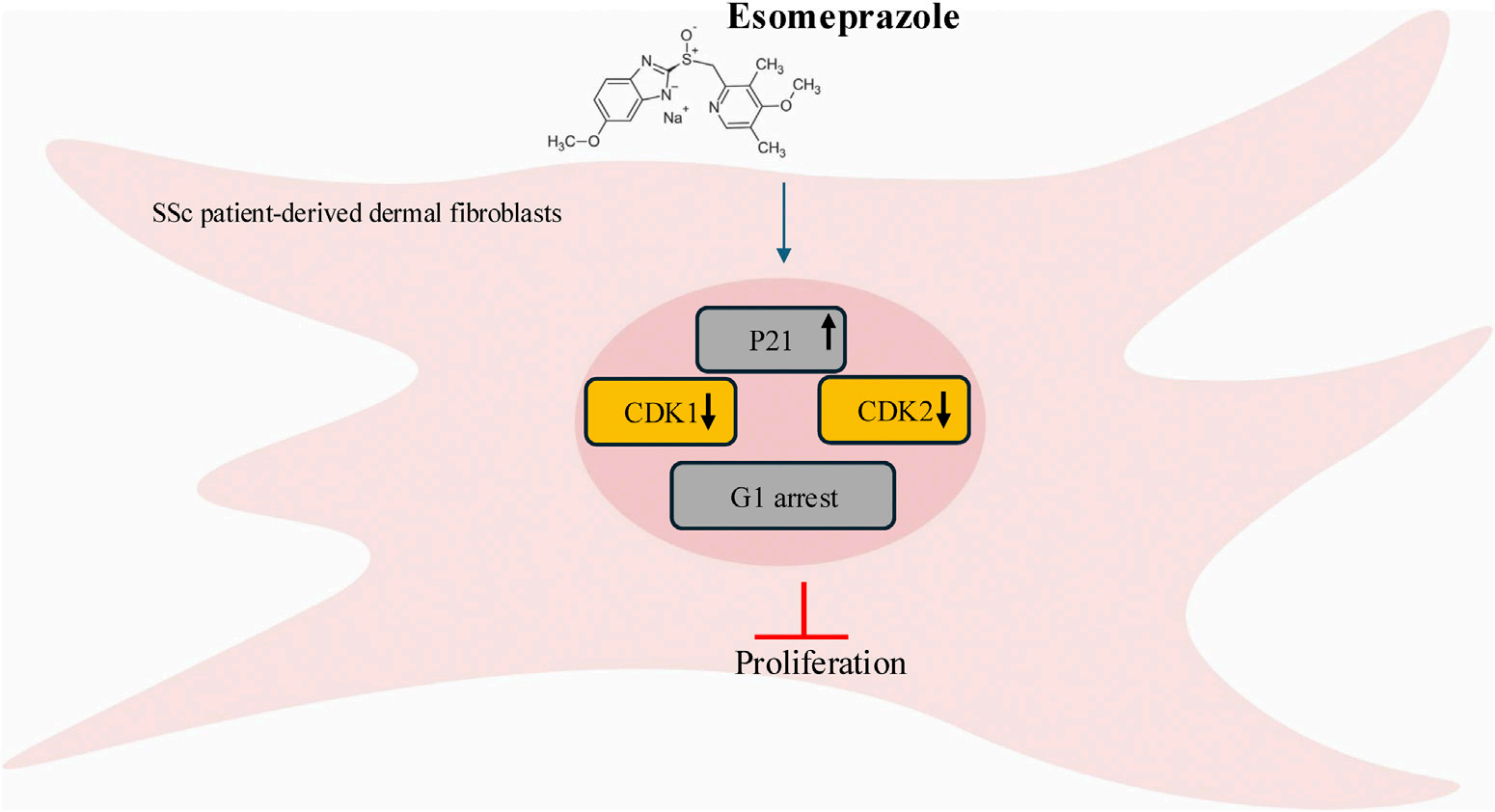
A cartoon modeling how esomeprazole controls the cell cycle. Esomeprazole upregulates p21 to downregulate CDK1 and CDK2 and induce cell cycle arrest to control the proliferation of scleroderma fibroblasts.

To explore the molecular mechanisms underlying the anti-proliferative effect of esomeprazole, we performed Western blot studies and observed a significant upregulation of p21 and downregulation of the cyclin-dependent kinases CDK1 and CDK2 following esomeprazole treatment (Figures 3a–d). The p21 protein is a direct CDK inhibitor that plays a critical role in regulating the cell cycle, in part by inhibiting the cyclin-CDK1/CDK2 complexes and preventing the G1-to-S phase transition (Uxa et al., 2021; Gartel et al., 1996; Schafer, 1998). The two kinases are key regulators of G1/S and G2/M transitions. The sustained upregulation of p21 over a 12–36-hour period suggests that esomeprazole activates CDK inhibition and G1 arrest pathways (Figure 5). Several studies have highlighted the central role of CDKs in cell cycle progression and their potential as therapeutic targets in hyperproliferative disorders such as cancer (Lapenna and Giordano, 2009; Roskoski, 2016; Bing et al., 2022).

Transcriptomic analysis further supports our findings by demonstrating that esomeprazole suppresses the expression of multiple genes involved in cell cycle regulation (Figure 4). For example, treatment with esomeprazole was found to downregulate CDK1 expression, confirming our low-throughput data showing that CDK1 is targeted by esomeprazole (Figure 3). In addition, cyclin B (CCNB) and Cell Division Cycle Protein 20 (CDC20) genes, which are essential for mitosis and spindle checkpoint activation (Shang et al., 2018; Hayward et al., 2019), were significantly downregulated by esomeprazole. The identification of DEGs in response to esomeprazole treatment underscores its broader impact on fibroblast biology, extending beyond mere regulation of the cell cycle. Future studies should focus on pathway enrichment analysis to identify specific esomeprazole-affected signaling networks and explore potential upstream regulators of the observed transcriptional changes.

Although our findings provide compelling evidence for the antiproliferative effects of esomeprazole in scleroderma fibroblasts, certain limitations should be acknowledged. First, the study was conducted primarily *in vitro*, and the relevance of these findings *in vivo* remains to be evaluated. Further mechanistic studies are warranted to determine how esomeprazole may influence ECM remodeling.

In conclusion, our study provides new evidence that esomeprazole exerts anti-proliferative effects on scleroderma fibroblasts by inducing cell cycle arrest through p21 upregulation and CDK1 and CDK2 downregulation (Figure 5). The RNA-seq data further support this observation by demonstrating broad transcriptional changes affecting proliferation-related pathways. Together, these findings support esomeprazole as a promising therapeutic candidate for the treatment of scleroderma.

## Data Availability

The original contributions presented in the study are publicly available. This data can be found here: https://www.ncbi.nlm.nih.gov/geo/query/acc.cgi?acc=GSE314222.
