# Supplementary material for: Esomeprazole inhibits proliferation of scleroderma fibroblasts via cell cycle regulation
Source: Front Pharmacol. 2026 Jan 6;16:1703115. doi: 10.3389/fphar.2025.1703115 (PMC12816232; doi:10.3389/fphar.2025.1703115)
Supplement: Supplementary file 1 [file DataSheet1.pdf]

Table S1. Downregulated cell cycle and proliferation-related genes (n=202) in scleroderma fibroblasts in response to esomeprazole treatment. RNA sequencing was performed on dermal fibroblasts (derived from two male donors with limited scleroderma disease) after treatment with esomeprazole at concentrations of 50 µM and 100 µM. The table summarizes significantly downregulated gene sets for each condition.

Symbol	Log2FC_50µm	p_adj_50µM	log2FC_100µM	p_adj_100µM
ANLN	-0.4127807045147340	0.6314158850629350	-0.849899590390167	0.0257740566219771
ANP32E	-0.4399726006820070	0.5645016523755960	-0.8677272260773990	0.02864452942574870
ASF1B	-0.4109655973712160	1.0	-0.8811020651857000	0.1657113267384140
ASPM	-0.6215497655198060	0.04466635265863680	-1.138879450782260	0.0003898249432469760
ATAD2	-0.3506792364933140	1.0	-0.7447426090667790	0.1466172095205020
ATAD5	-0.4385329154333000	1.0	-0.8101021798744220	0.9018024208998130
AUNIP	-0.4478185179209490	1.0	-1.146618900672110	0.2912672150402900
AURKA	-0.469592650021676	0.4554011591778520	-0.8629498376063840	0.03695969795557250
AURKB	-0.5424311981582270	0.4351627189414360	-1.011932250447670	0.02597608102333240
BIRC5	-0.569627210391899	0.1250201019926350	-0.956310470662606	0.009544689468971950
BRCA2	-0.4175840164743650	1.0	-0.8369661386961270	0.2683066217114190
BUB1	-0.4303750968085770	0.7374127793826170	-0.8533985442839980	0.05589744822891580
BUB1B	-0.5894046241068130	0.1580413506475360	-1.052960629984910	0.005974602571025640
CBX5	-0.4237386120118830	0.590399809735431	-0.6283379774523450	0.2912672150402900
CCNA2	-0.4024155013448770	0.689439119547312	-0.8238987823214590	0.03645095727755490
CCNB1	-0.5421741930206770	0.1577182119397820	-0.9347431951865840	0.00988645513711891
CCNB2	-0.4264217930852470	0.7841565407984760	-0.9082713351134820	0.03318859193101570
CCNB3	-2.947831146222380	1.0	-	0.7025756971200820
CCNE2	-0.4538423055487190	1.0	-0.9408887874097760	0.7115904952128950
CCNF	-0.5525423543390770	0.3075445680785590	-0.9802890143505570	0.02341364716770470
CD24	-0.6794449043757370	0.6586262317968260	-0.8417347287376610	0.5391741538903090
CD4	-	0.5570794542052120	-inf	0.7025756971200820
CDC20	-0.5859017842724230	0.1042212239068890	-1.208194549734370	0.0002132839586886210
CDC25C	-0.3124864563031770	1.0	-0.7769315092271020	0.8334233489318490
CDC45	-0.6390100399764990	0.8087455804316270	-0.9787585384661430	0.2842614983823320
CDC6	-0.6665686577210600	0.291825403973437	-0.6290037070317750	0.8334233489318490
CDCA2	-0.4828171731645190	0.6857844278949930	-0.8734952605533460	0.09806892050407670
CDCA3	-0.413284675504554	1.0	-0.8101494016161050	0.1466172095205020
CDCA5	-0.3747535289947510	1.0	-0.7424061752848540	0.2358056394008030
CDCA8	-0.5155257361717770	0.354220878679009	-0.9379961864455520	0.0257740566219771
CDK1	-0.3280445660413910	1.0	-0.7550358523648180	0.1264844225513030
CDKN2C	-0.5578322053471500	0.2402833514435320	-0.9121508511171710	0.03382629719994870
CDKN2D	-0.3816260039653560	1.0	-0.7217193036092580	0.6863408452976370
CDKN3	-0.4552215552148480	1.0	-1.125002549536410	0.01969625212513310
CDT1	-0.7521553663872080	0.1544263104675810	-1.139874078295490	0.0257740566219771

Symbol	Log2FC_50µm	p_adj_50µM	log2FC_100µM	p_adj_100µM
CENPA	-0.3616370013888340	1.0	-0.8020921911542500	0.3296899392810330
CENPE	-0.4386264347887940	0.5690813283736120	-0.8695277795431610	0.02817433242260680
CENPF	-0.5283684608354520	0.1714402652489080	-0.9563930402961730	0.006695311226659880
CENPI	-0.4850829858006230	1.0	-0.8043467398972860	0.4325084778036330
CENPK	-0.4838316239033730	0.9797853145690090	-0.8824366270638280	0.187095156034131
CENPM	-0.37263534944001	1.0	-0.7626249638781000	0.6666854879332920
CENPN	-0.3641407512541270	1.0	-0.6477145651971900	0.3664141677721700
CENPO	-0.4169344104452730	0.9393507940886140	-0.6794103108510180	0.3513784291909400
CENPU	-0.3461589956426050	1.0	-0.6764927895675720	0.4503911063548030
CENPW	-0.309391771133025	1.0	-0.6375179211063400	0.9553631225501570
CEP55	-0.3323517621284610	1.0	-0.7703022268404800	0.09658210838743590
CHAF1A	-0.6256336656483300	0.2095078489773480	-0.8673934397448940	0.1093439966002830
CHURC1-FNTB	-0.1590355536604450	1.0	-2.281925705244840	0.03666999746934170
CIT	-0.5822641070800330	0.1482973151299420	-0.9398879135483410	0.01863634686306930
CKAP2	-0.2885627676819750	1.0	-0.7443143220507340	0.09914084593808950
CLSPN	-0.5874289035203860	0.3456481455830430	-0.5706826757402440	0.8844794973134480
COMMD3-BMI1	-inf	0.01560015695548810	-inf	0.04166567595910430
CSF2RB	-0.5026484833233660	1.0	-1.432485381820230	0.3847869288285640
CXCL12	-0.5563739499129640	0.1250201019926350	-0.6591807490036110	0.2166533426948550
DACT1	-0.3207814310970140	1.0	-0.6188589115042840	0.5195016917159380
DBF4	-0.3254921661279400	1.0	-0.7559060057639770	0.3513784291909400
DBF4B	-0.4176567279190700	1.0	-0.6047046966716990	1.0
DDX11	-0.4118288038851740	1.0	-0.6451242730040690	0.9656871920258430
DEPDC1	-0.482902857012363	0.4262929025793670	-0.9912821888899310	0.01043814668403430
DIAPH3	-0.3261830310834160	1.0	-0.8961142463254930	0.03271799383350230
DLGAP5	-0.528016477920815	0.2400052026125920	-1.027827882633200	0.005386778462874480
DNMT1	-0.4723260439801930	0.3460654162115160	-0.8071819598417320	0.05041986081358690
DOCK2	-0.4079856700838570	1.0	-0.6751037705599480	0.7025756971200820
DTL	-0.6137257357844550	0.2906553267523500	-0.8298303181529030	0.1791127354755450
DTYMK	-0.3878926691837050	1.0	-0.7353421375124640	0.1974888828122240
E2F7	-0.4807438543256980	0.7743039239113410	-0.7989966914947810	0.2027686424771090
E2F8	-0.3154557030264460	1.0	-1.021109124660870	0.5629403617927370
ECT2	-0.2724824090441220	1.0	-0.7230470933308640	0.1466172095205020
EDN1	-0.8410711459035570	0.1042212239068890	-1.013108946817040	0.1089796288729870
EME1	-0.009996843405597520	1.0	-0.7732290987517540	1.0
EPGN	-1.154282023689810	1.0	-inf	0.4853492676546150
ESCO2	-0.3474202464125640	1.0	-0.9878766429329180	0.2385909053818800
ESM1	-0.7709533841383050	1.0	-1.576668970805980	0.9553631225501570
ESPL1	-0.5681413317713730	0.3114432881690980	-0.8229227584398150	0.1321539089454120

Symbol	Log2FC_50µm	p_adj_50µM	log2FC_100µM	p_adj_100µM
ETV5	-0.4122289332623660	0.9655691543515020	-0.6642320026908140	0.4037084564182680
EZH2	-0.1355265492727740	1.0	-0.6982144578899690	0.5305091262568650
FAM83D	-0.4355800991559900	0.7844489518055230	-1.002899266643150	0.01616348173968230
FANCA	-0.6672390605544650	0.2902966445598900	-0.7730233081457580	0.3807294560881540
FANCD2	-0.4663706538371500	0.9820828429670260	-0.9825401790085690	0.07468690049672420
FBXO5	-0.4498589225679650	1.0	-0.9180187019642300	0.1243424266985320
FOXM1	-0.5309880208744410	0.1736145835966470	-0.7467972466710240	0.09914084593808950
GATA2	-0.7537692388985620	0.1644143794823830	-0.9336863332416340	0.1466172095205020
GINS3	-0.6229005631734980	1.0	-0.4156770941336710	1.0
GINS4	-0.705030755043808	0.1384390419437800	-0.845133653325478	0.1666226024752670
GOLGA8K	-inf	0.3361273415105270	-inf	0.4853492676546150
GPSM2	-0.2604368482056930	1.0	-0.71120044218046	0.3161701015928870
GRB14	-inf	1.0	0.4550398889213630	1.0
GTSE1	-0.5742283233522780	0.1920349700823710	-1.030700601700680	0.0087184277857718
HAUS8	-0.3856274702734120	1.0	-0.7303003883977990	0.8708010417510800
HELLS	-0.3873989068263900	1.0	-0.7872955242445950	0.7969727231424530
HJURP	-0.3209359131425390	1.0	-0.8691552775178450	0.05085393121922490
HMGB2	-0.3453522788135800	1.0	-0.7177004512660850	0.1265340976369400
HMGB3	-0.5482295954318580	0.3101036150947420	-0.8089716216168240	0.1219474863878960
HMGN2	-0.4046093292427190	0.6699136002932410	-0.591433774778322	0.3847869288285640
IL11	-0.8409465393185000	0.2117697553748120	-1.107818425092930	0.1219474863878960
INCENP	-0.4113964579797870	0.856761506514583	-0.8027836606647230	0.1051432689913320
INHBA	-0.3653618205068000	1.0	-0.7797955950483240	0.125158368338203
IQGAP3	-0.4298149147064740	0.5734248869332550	-0.8682764139546490	0.02537707952193380
KIF11	-0.4769213098652090	0.3745896012112930	-0.8933056008067940	0.02330303814569230
KIF14	-0.6313765414820830	0.09402161386653130	-1.117041320503060	0.002500279826521880
KIF15	-0.4529583012067890	1.0	-0.9203139088849990	0.1265340976369400
KIF18A	-0.1544638263541410	1.0	-0.6403983215300910	0.5968334569672730
KIF18B	-0.5891211641841400	0.3361273415105270	-1.170564596297140	0.007713005950140910
KIF20A	-0.5728254664701900	0.1187685572032920	-1.047010304943000	0.002571899143595420
KIF20B	-0.50157491633282	0.3460654162115160	-0.9112185764217970	0.0257740566219771
KIF22	-0.3582573032769310	1.0	-0.7523339671276230	0.1666575379776690
KIF23	-0.4397726386172110	0.5009029679056260	-0.8973404631412270	0.0170546225370416
KIF2C	-0.5718470462301820	0.1596119241589650	-0.9294259113389250	0.01966479706477760
KIF4A	-0.3789929904496460	1.0	-0.9903003705210070	0.01587895828276480
KIFC1	-0.4933706697353560	0.4093252731133340	-0.91738768797364	0.02597608102333240
KNTC1	-0.2774005668924750	1.0	-0.657157016083364	0.4503911063548030
KPNA2	-0.3338957200510980	1.0	-0.7325929255860440	0.1063632646432930
LIG1	-0.7071636664913650	0.1031223213046040	-0.8039133401006140	0.1797545233646430
LMNB1	-0.5525323793679200	0.1930751238990240	-0.9864022149312050	0.00988645513711891
MAD2L1	-0.3289488372591920	1.0	-0.7276642437134370	0.277356800024057

Symbol	Log2FC_50µm	p_adj_50µM	log2FC_100µM	p_adj_100µM
MCM2	-0.5426992897491390	0.3499801162343760	-0.7624758121403330	0.1913545951579890
MCM3	-0.4679905675946890	0.6174014087391930	-0.5942410138498550	0.584961629170741
MCM5	-0.543070597042673	0.2402833514435320	-0.7236390098570300	0.1833286484750950
MCM7	-0.4262352606830980	0.6174014087391930	-0.8099205111004670	0.05439672639076740
MDC 1.00	-0.3688549405669590	1.0	-0.6056878642775130	0.5150443535554730
MIS18A	-0.5858419788813170	0.8175024202282130	-0.8424539876558040	0.4287117927623590
MKI67	-0.5868467323383780	0.07992226232554230	-1.103956529735640	0.0006400760839907580
MMS22L	-0.3873422722499700	1.0	-0.5981687049119560	0.9569099076778740
MSH5	-1.298738308081510	0.2132473418866840	-0.2743788961914950	1.0
MXD3	-0.743121994699053	0.1158747197243620	-1.064599821304830	0.03145120891409740
MYBL2	-0.5965277599146170	0.2149636028485690	-0.98465966459175	0.0257740566219771
NCAPD2	-0.4471889304921070	0.4554011591778520	-0.7447576134894520	0.1031470362918060
NCAPG	-0.4465685795204410	0.590399809735431	-0.8587644857807550	0.04099695975665580
NCAPG2	-0.4405238206849300	0.6156000929259200	-0.7527890148889760	0.125158368338203
NCAPH	-0.6322760933535350	0.1930751238990240	-0.9117947369941580	0.07148014830365250
NDC1	-0.3208855144108180	1.0	-0.692336326120572	0.3335264016081390
NDC80	-0.3507157522524310	1.0	-0.9527452681292210	0.03141112192474250
NEDD9	-0.2397982787590620	1.0	-0.8536263065570770	0.540701637187067
NEK2	-0.4235984811617110	0.9982555420227070	-0.8848402507887410	0.09108441550730370
NES	-0.6589229838586010	0.322125409685442	-0.7763314432191780	0.3834056950281140
NET1	-0.2655235692409230	1.0	-0.6450536132738780	0.5055174840504220
NOG	-0.6744362287828690	0.1714402652489080	-0.7311581244072840	0.3597532047224520
NUF2	-0.4560488240123190	0.9606456453598830	-0.7777403012098380	0.2683066217114190
NUSAP1	-0.361621178894417	1.0	-0.7397462159401590	0.1367974018623570
ORC6	-0.4490252892509430	1.0	-0.7746083484178840	0.4574606720394320
PBK	-0.4368529187644250	0.7841565407984760	-0.8184434661434620	0.1065260314858990
PDCD1LG2	-0.4729665741659670	0.6376130408310280	-0.9731085258484190	0.0252964666794374
PIF1	-0.2617467210583620	1.0	-0.6874717555463900	0.8334233489318490
PKMYT1	-0.623505883725803	0.2795695695554490	-0.8374020192981690	0.1791127354755450
PLK1	-0.603392049875018	0.08813185112952300	-1.085203084739300	0.001853107693298090
PLK4	-0.40275031638894	1.0	-0.9197500203516260	0.1118984395066590
PNP	-0.486966861091097	1.0	-0.6747975487212190	1.0
POC1A	-0.265136978271246	1.0	-0.7170914060246350	0.4503911063548030
POLE	-0.2715142222814660	1.0	-0.6395424048689940	0.4425143114844650
POU3F2	-1.151176112821990	1.0	-2.817978605485050	0.05695923302105390
PRC1	-0.476981441988905	0.3101036150947420	-0.9345028746391690	0.008832465336129530
PSMC3IP	-0.2883798395321310	1.0	-0.7815663916347860	0.7908663952804950
PSRC1	-0.2623417695472640	1.0	-0.582630529044692	0.8334233489318490
PSTPIP1	0.3583296328066620	1.0	-inf	0.1041532569259090
PTGS2	-1.75640507556915	0.005823968161135790	-1.96781711481678	0.01084870812046370

Symbol	Log2FC_50µm	p_adj_50µM	log2FC_100µM	p_adj_100µM
PTH1R	-0.3348542693316310	1.0	-inf	0.3296899392810330
PTTG1	-0.4109274157203660	0.9393507940886140	-0.8224094189212110	0.1050361252329990
RACGAP1	-0.435981798883488	0.5236627275974950	-0.7626750343993000	0.08279086862142760
RAD51	-0.350837077034424	1.0	-0.6819883605561500	0.7258749513386000
RAD54L	-0.5466918351235220	1.0	-0.8093466210797120	0.5815646512533820
RANBP1	-0.4976302286099980	0.3309019891862950	-0.7071741638101190	0.1797545233646430
REEP4	-0.4628447693809760	0.6699136002932410	-0.6194100602385180	0.5113652720597010
RFC3	-0.3136251264813790	1.0	-0.8066079766414690	0.3597532047224520
RGCC	-0.9187686806199210	1.0	-1.176808999430630	0.9607711586603200
RNASEH2A	-0.4434033571220870	1.0	-0.6769254512871420	0.540701637187067
RRM2	-0.6393758571965110	0.04692448289426790	-1.016598900544990	0.00479357409631617
RTEL1	-0.2078280941320540	1.0	-0.756396118042845	0.246635582950791
RTTN	-0.2269273255215760	1.0	-0.5894347402192420	0.8780483564800300
SAPCD2	-0.4553161981075840	1.0	-1.180263730585540	0.01966479706477760
SERPINB7	-0.232794051868334	1.0	-0.5985147164355930	1.0
SKA1	-0.3040638772181820	1.0	-0.7360182401855280	0.5141567633632140
SKA3	-0.5363044657406910	0.4779782720345260	-1.129922611799790	0.0087184277857718
SLX4	-0.3932980045843690	1.0	-0.7349892408140210	0.6172425080437380
SMC2	-0.2994634094424460	1.0	-0.5906368994371490	0.4853492676546150
SMC4	-0.4784650041014070	0.3075445680785590	-0.7301067056603740	0.1082195478295770
SOX9	-0.8667874258663220	0.1066194379664010	-0.9118140768475160	0.2485649493737590
SPAG5	-0.4723330314627430	0.4351627189414360	-0.8162816497423860	0.0636030367817106
SPC25	-0.183647544087939	1.0	-0.8765483170165690	0.3507505596318870
SPDL1	-0.4097011303367850	0.6983706829372280	-0.8126636975056080	0.05098160317315950
STIL	-0.2344781092781290	1.0	-0.7673525324150850	0.2566935806813850
STMN1	-0.2705044962733140	1.0	-0.6562836444610660	0.2140027084993100
TACC3	-0.4320774348249130	0.588357951981607	-0.8147472544736470	0.05085393121922490
TCF19	-0.4636707256911280	0.5319824450592500	-0.7928466021572940	0.09840816490329920
TGFB2	-0.5452277164577450	1.0	-1.060601551295890	0.5660995941584000
TICRR	-0.5501876627904580	0.7743039239113410	-0.5912157020395100	1.0
TK1	-0.5759657970496200	0.1384390419437800	-0.9455084422502660	0.01383369381708410
TMPO	-0.4454564245636810	0.4554011591778520	-0.8422225323670700	0.030133996255349
TOP2A	-0.3538769707509710	1.0	-0.8529399025598500	0.02597608102333240
TPX2	-0.4472391327451090	0.4262929025793670	-0.9554327725350260	0.006695311226659880
TRIP13	-0.5179496842968980	0.3981560249303810	-1.081270993509910	0.006695311226659880
TROAP	-0.3435484231772360	1.0	-0.9117646961182510	0.1095599390085140
TTK	-0.2744362510955210	1.0	-0.7909888966823230	0.1466172095205020
UBE2C	-0.43678697623713	0.6699136002932410	-0.9334523344984960	0.0215568105507509
UBE2S	-0.5648650384081520	0.1135639811948280	-0.9356664158763330	0.009544689468971950
UBE2T	-0.3044223731176710	1.0	-0.7617715001517850	0.4503911063548030
UHRF1	-0.6431283714012000	0.08394568259264150	-0.9570860149375780	0.01880131444243130

Symbol	Log2FC_50µm	p_adj_50µM	log2FC_100µM	p_adj_100µM
WDR62	-0.4100291068973170	1.0	-0.8968037838702870	0.1073879532665600
WDR76	-0.3907556032524700	1.0	-0.6236590725459520	0.9957435911380980
WNT4	-inf	0.8087455804316270	-0.834466728273622	1.0
XRCC2	-0.1829427203544500	1.0	-1.106315998847460	0.4738710689768960
ZWILCH	-0.3483095537728970	1.0	-0.6474750217045720	0.3598537637097500
ZWINT	-0.4622769980900330	0.7644884516305280	-0.8153813604129800	0.1421262870085440
