# Supplementary material for: Esomeprazole inhibits proliferation of scleroderma fibroblasts via cell cycle regulation
Source: Front Pharmacol. 2026 Jan 6;16:1703115. doi: 10.3389/fphar.2025.1703115 (PMC12816232; doi:10.3389/fphar.2025.1703115)
Supplement: Supplementary file 2 [file Image1.pdf]

Supplemental Figure S1

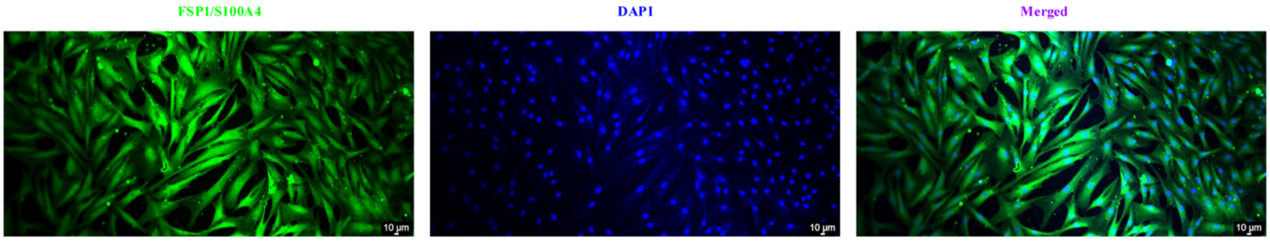

Figur S1. IF-Staining of Patient-derived fibroblasts using FSP1

Immunofluorescence analysis of patient-derived dermal fibroblasts fixed with 4% PFA, using fibroblast-specific protein antibody S1004A/FSP1 (66489-1-Ig, Proteintech) and Alexa Fluor 488 (A-10680, Invitrogen). Nuclei were counterstained with 4',6-diamidino-2-phenylindole (DAPI) (Sigma, cat # D9542). Images were captured using a Leica Stellaris 5 confocal microscope.
